# A novel DNA damage repair-related signature for predicting prognositc and treatment response in non-small lung cancer

**DOI:** 10.3389/fonc.2022.961274

**Published:** 2022-11-04

**Authors:** Ling Li, Bao-jia Zou, Juan-zhi Zhao, Jia-bi Liang, Zi-yue She, Wen-ying Zhou, Si-xiao Lin, Lin Tian, Wen-ji Luo, Fa-zhong He

**Affiliations:** ^1^ Department of Pharmacy, The Fifth Affiliated Hospital, Sun Yat-sen University, Zhuhai, Guangdong, China; ^2^ Department of Hepatobiliary Surgery, The Fifth Affiliated Hospital of Sun Yat-sen University, Zhuhai, China; ^3^ Department of Central Laboratory, The Fifth Affiliated Hospital, Sun Yat-sen University, Zhuhai, Guangdong, China; ^4^ Department of Quality Control, Zhuhai People’s Hospital, Zhuhai Hospital Affiliated with Jinan University, Zhuhai, Guangdong, China

**Keywords:** non-small lung cancer (NSCLC), tumor microenvironment, DNA damage repair-based prognostic signature, NSCLC classification, biomarker

## Abstract

DNA damage repair (DDR) is essential for maintaining genome integrity and modulating cancer risk, progression, and therapeutic response. DDR defects are common among non-small lung cancer (NSCLC), resulting in new challenge and promise for NSCLC treatment. Thus, a thorough understanding of the molecular characteristics of DDR in NSCLC is helpful for NSCLC treatment and management. Here, we systematically analyzed the relationship between DDR alterations and NSCLC prognosis, and successfully established and validated a six-DDR gene prognostic model *via* LASSO Cox regression analysis based on the expression of prognostic related DDR genes, *CDC25C*, *NEIL3*, *H2AFX*, *NBN*, *XRCC5*, *RAD1*. According to this model, NSCLC patients were classified into high-risk subtype and low-risk subtype, each of which has significant differences between the two subtypes in clinical features, molecular features, immune cell components, gene mutations, DDR pathway activation status and clinical outcomes. The high-risk patients was characterized with worse prognosis, lower proportion and number of DDR mutations, unique immune profile and responsive to immunetherapy. And the low-risk patients tend to have superior survival, while being less responsive to immunotherapy and more sensitive to treatment with DNA-damaging chemotherapy drugs. Overall, this molecular classification based on DDR expression profile enables hierarchical management of patients and personalized clinical treatment, and provides potential therapeutic targets for NSCLC.

## Introduction

Non-small cell lung cancer (NSCLC) is a malignant tumor with a high clinical incidence and frequent cause of cancer death (1, 2). Currently, the main methods for treating NSCLC include surgery, chemotherapy, radiotherapy, targeted therapies, immunotherapies, or combination cancer therapy (3). Despite significant progress in NSCLC diagnostic and therapeutic techniques, the outlook for NSCLC patients is still poor. So, a reliable predictive biomarker is urgently needed to identify patients at the highest risk in order to direct more personalized treatment and prevention for NSCLC. Thus far, a variety of studies utilizing genomic, transcriptomic, and proteomic data have recommended molecular and immune classifications of NSCLC (4, 5). These subtyping strategies provide effective targeted therapy options for NSCLC by revealing the molecular phenotypes. Nonetheless, the molecular mechanisms involved with the poor outcome of NSCLC remain unclear. Genomic instability is one of the most remarkable characteristics of cancer cells. Many cancer-related risk factors, such as smoking, ionizing radiation or exogenous anti-cancer chemotherapeutic drugs, can cause DNA damage. DDR is vital for maintaining the stability of the human genome by regulating the cell cycle, chromatin remodeling, metabolism, immunogenicity, and apoptosis. DDR defects are common among advanced cancers; the functional loss of DDR may result in the onset and progression of cancer, and it may change treatment effectiveness (6, 7). Based on role of the DDR pathway in cancer, targeting the DDR pathway is a promising treatment option for cancers. For instance, the classic DDR pathway drug, Olaparib, which is a poly (ADP-ribose) polymerase (PARP) inhibitor, is a targeted drug in the base excision repair pathway (BER) (8). Thus, an in-depth and precise analysis of the molecular changes caused by DDR can aid in the understanding of the development mechanisms of cancers and contribute to explore new therapeutic targets.

Recently, a study based on DDR activity status has characterized hepatocellular carcinoma (HCC) patients into two classes, namely, DDR-activated and DDR-suppressed subtypes, by mRNA expression profiling of DDR Genes, implying that each DDR subtype has distinct clinical and molecular characteristics (9). HCC patients with the DDR-activated subtype exhibit aggressive clinical behavior and a poor prognosis, whereas those with the DDR-suppressed subtype have a positive prognosis (9); in addition, the immune profiles and immunotherapy responses of the two DDR subtypes also differ (9). Another recent study has reported that immune checkpoint inhibitors (ICIs) provide longer OS for HCC patients who have high expression of DDR-related genes (10). Therefore, DDR-based molecular classification provides a fundamental basis for implying clinical outcomes and selection of strategies for HCC treatment. However, a thorough analysis of DDR-relevant molecular classification is lacking in NSCLC.

In this study, we explored the relationships of transcriptional profile alteration of DDR genes with the prognostic value and immune infiltration in NSCLC. Firstly, we identified 17 prognostic DDR-related genes from 222 DDR genes *via* a univariate Cox proportional hazards model. Based on the 17 prognostic DDR-related genes, we successfully identified two NSCLC subtypes *via* unsupervised consensus clustering. The two NSCLC subtypes differ in clinical outcomes and molecular characteristics. Based on prognostic DDR-related gene analysis, we further demonstrated heterogeneity between the subtypes and proposed a new method to predict immune treatment outcomes. In the present study, we discuss the DDR alterations in NSCLC, which may help guide immunotherapy and determine the prognosis of NSCLC patients.

## Materials and methods

### NSCLC data collection and processing

The transcriptome data from RNA sequencing (RNA-seq) and related clinical information, including gender, age, stage, and TNM, of 986 NSCLC patients were acquired from The Cancer Genome Atlas (TCGA) database *via* the University of California Santa Cruz (UCSC) Xena database (https://xenabrowser.net/datapages/). The mutation information of these NSCLC samples was downloaded using the RTCGA and TCGA biolinks packages in R (11). Firstly, the expression profiles of the two TCGA data sets (LUAD and LUSC) were combined, and more than 70% of the genes not be detected (NA) were filtered. In R, the SVA package was used to remove heterogeneity, and the expression profiles of 27875 genes ×986 samples were obtained in the training set. Expression profiles for genes were transformed into log2 [(FPKM) +1] for further analysis in the training set. Validation testing was conducted using the GSE68465 dataset downloaded from the Gene Expression Omnibus (GEO) database, which included 442 NSCLC samples (12). For further analysis, gene expression profiles were converted into log2 (normalized read count + 1). The clinical characteristics of these NSCLC patients are listed in **Supplementary Table 1**.

### Prognostic DDR-related gene recognition

First, we collected 276 DDR pathway-related genes and 80 DDR pathway key genes from previous studies by TCGA DDR-AWG (13, 14). Based on MSigDB v5.0 and DDR pathway knowledge, these genes were compiled. In total, 222 of the 276 DDR-related genes were expressed in *both datasets* (training set and verification set). Based on the training set, prognosis significance and the hazard ratio (HR) of 222 DDR- related genes were conducted utilizing Cox regression analysis using the survival package in R. We selected genes with a p<0.05 as prognostic DDR-related genes. To explore the biological functions of these prognostic DDR-related genes, Gene Ontology (GO) and Kyoto Encyclopedia of Genes and Genomes (KEGG) analyses were conducted *via* the clusterProfiler package in R.

### NSCLC subclass identification and validation

Based on the above analysis, we obtained 17 prognostic DDR-related genes that have prognostic potential to identify NSCLC subclasses. The expression profiles of the prognostic DDR genes were obtained for the above samples (**Supplementary Table 1**). Unsupervised consensus clustering was used to discover intrinsic NSCLC subtypes in the training set and testing set with the following parameters: *reps* = 100, *clusterAlg* = “km”, *distance* = “Euclidean” (15). The optimal cluster number (K) of unsupervised consensus clustering was determined according to the proportion of ambiguous clustering (PAC) method (16). The unsupervised clustering analysis identified two principal clusters (cluster 1 and cluster 2) for NSCLC samples in the training set. Using the same procedure, we validated the results in the testing set. In addition, principal components analysis (PCA) was used to demonstrate the existence of the clusters and assess their reproducibility in the two independent cohorts. The consensus clustering analysis was performed by the ConsensusClusterPlus package in R (15). The difference in OS between the two subgroups was compared *via* the Kaplan-Meier method with log-rank testing.

### Differentially expressed gene and GO analyses of NSCLC subclasses

To explore biological features between the two NSCLC subtypes, gene set enrichment analysis (GSEA) was employed to identify significantly enriched GO terms and KEGG subsets from canonical pathways (c5.go.bp.v7.2.symbols.gmt). Differential expressions were then identified using the limma package in R. The cutoffs of |logFC|>0.3 and FDR<0.05 were utilized for GO terms and pathway sets to establish statistical significance. Moreover, the Wilcoxon rank-sum test was used to test for differences in the mean expression of 80 key DDR pathway genes between the NSCLC subtypes. To better clarify the two NSCLC subclasses, Fisher’s exact test was performed to examine the relationship between the two NSCLC subclasses and clinical features.

### Mutation differences of NSCLC subclasses

To distinguish biologically significant copy number variation in all samples, the GISTIC algorithm (17) was used in the SNP6 Copy Number segmented profiles using the *MutSigCV* module in GenePattern (https://cloud.genepattern.org/gp/pages/index.jsf). The q-value cutoff for peak significance was 0.05, and the confidence level for determining the peak interval was 0.90.

### Immune infiltration estimation and immunotherapy prediction of NSCLC subclasses

To investigate the differences in molecular characteristics and biodiversity patterns between the NSCLC subclasses, the ESTIMATE algorithm was used to assess immune infiltration, including immune score, stromal score, and tumor purity (18). Single-sample gene set enrichment analysis (ssGSEA) was then used to measure the level of immune infiltration in a sample by analyzing the expression levels of immune cell-specific markers (19). The marker genes for 28 types of immune cells were collected from a previously published research (20). The c5.go.bp.v7.2.symbols.gmt gene set was downloaded from the MSigDB database (https://www.gseamsigdb.org/gsea/index.jsp) for the GSEA algorithm (21), and the Wilcoxon rank-sum test was then used to estimate immune profile differences between the subtypes. Furthermore, compared the clinical effects of immune checkpoint therapy between NSCLC subclasses *via* the TIDE tool (http://tide.dfci.harvard.edu/) (22, 23). In addition to the differential analysis described above, a heatmap was generated to visualize the data.

### Prognostic DDR-related gene signature development and validation for NSCLC

Because too many genes make clinical application difficult, it is necessary to identify the crucial prognostic genes and construct a prognostic model for NSCLC subtypes. Based on the nine intersection genes between the prognosis-related DDR genes and differentially expressed genes (DEGs), we developed a six DDR-related gene prognostic model according to the individual risk score as follows:


$$Risk\;score=\overset{\;}{\underset{\sum }{\;}}\left(coefficient\times expression\;of\;signature\;gene\right)$$

The effectiveness of the signature was determined using PCA. Kaplan-Meier analysis was then used to compare OS between patients with low and high risk. Lastly, univariate and multivariate analysis assessed the prognostic values of clinical information (age, gender, stage, T, N, M and *EGFR* mutation) and gene expression with OS.

### Quantitative real-time PCR

Total RNA was extracted *via* Trizol (TRIzol, Invitrogen), and reverse transcription PCR was then performed using reverse transcription kits (Takara, RR047A). The PCR assays were performed according to the manufacturer’s instructions (Takara, RR091A). *GAPDH* was used as reference genes. The relative mRNA expression levels were quantified with the 2^−ΔΔCt^ method and determined with reference to *GAPDH* mRNA levels. The primer sequences are listed below: *CDC25C*-Forward Primer: ATGACAATGGAAACTTGGTGGAC; *CDC25C*-Reverse Primer: GGAGCGATATAGGCCACTTCTG; *GAPDH*-Forward Primer: ATGACAATGGAAACTTGGTGGAC; *GAPDH*-Reverse Primer: GGAGCGATATAGGCCACT TCTG.

### Immunohistochemical analysis

The data from immunohistochemistry analysis of the selected DDR-related factors (*CDC25C*, *NEIL3*, *H2AFX*, *NBN*, *XRCC5* and *RAD1*) in normal lung and NSCLC tumor tissues were obtained from the Human Protein Atlas (HPA) database (https://www.proteinatlas.org/).

### Cell culture and lentiviral shRNA knockdown of CDC25C

The A549 human NSCLC cancer cell line was obtained from the Cell Bank of the Shanghai Chinese Academy of Sciences (Shanghai, China) and cultured in RPMI 1640 cell medium (Gibco) supplemented with 10% FBS, 100 µg/ml streptomycin, and 100 U/ml penicillin. Cells were seeded in 6-well plates (1*10^5^ cells per well) and allowed to reach 80% confluence. Cells were then transfected with lentiviral vectors containing *CDC25C* small hairpin RNAs (shRNAs) designed by Shanghai Genechem Co., Ltd. (Shanghai, China). Two *CDC25C* shRNAs and a control shRNA were used as follows: shRNA1, 5′-CCGGGTCCCATTACTACTGTTCCAACTCGAGTTGGAACAGTAGTAATGGGACT-3′; shRNA2, 5′-CCGGGCCTTGAGTTGCATAGAGATTCTCGAGAATCTCTATGCAACTCAAGGCTTTTTG-3′; and shRNA-NC, 5′-CCGGTTCTCCGAACGTGTCACGTCTCGAGACGTGACACGTTCGGAGAATTTTTG-3′.

### Statistical analysis

Kaplan-Meier analysis and log rank tests were conducted to assess survival. Comparisons between groups were performed using Wilcoxon and t-tests. The Hazard-ratios (HR) and 95% confidence intervals (CIs) were estimated utilizing univariate and multivariable Cox regression model. In the graphical displays, NS denotes P>0.05, * indicates P ≤ 0.05, ** represents P ≤ 0.01, *** indicates *P*≤ 0.001, and **** indicates *P ≤* 0.0001. The mutation map generated the maftools package in R shows the mutation landscape of patients of different groups. P<0.05 was considered statistically significant and all statistical tests were two sided.

## Results

### Identification of two NSCLC subclasses based on prognostic DDR-related genes

**Figure 1:**
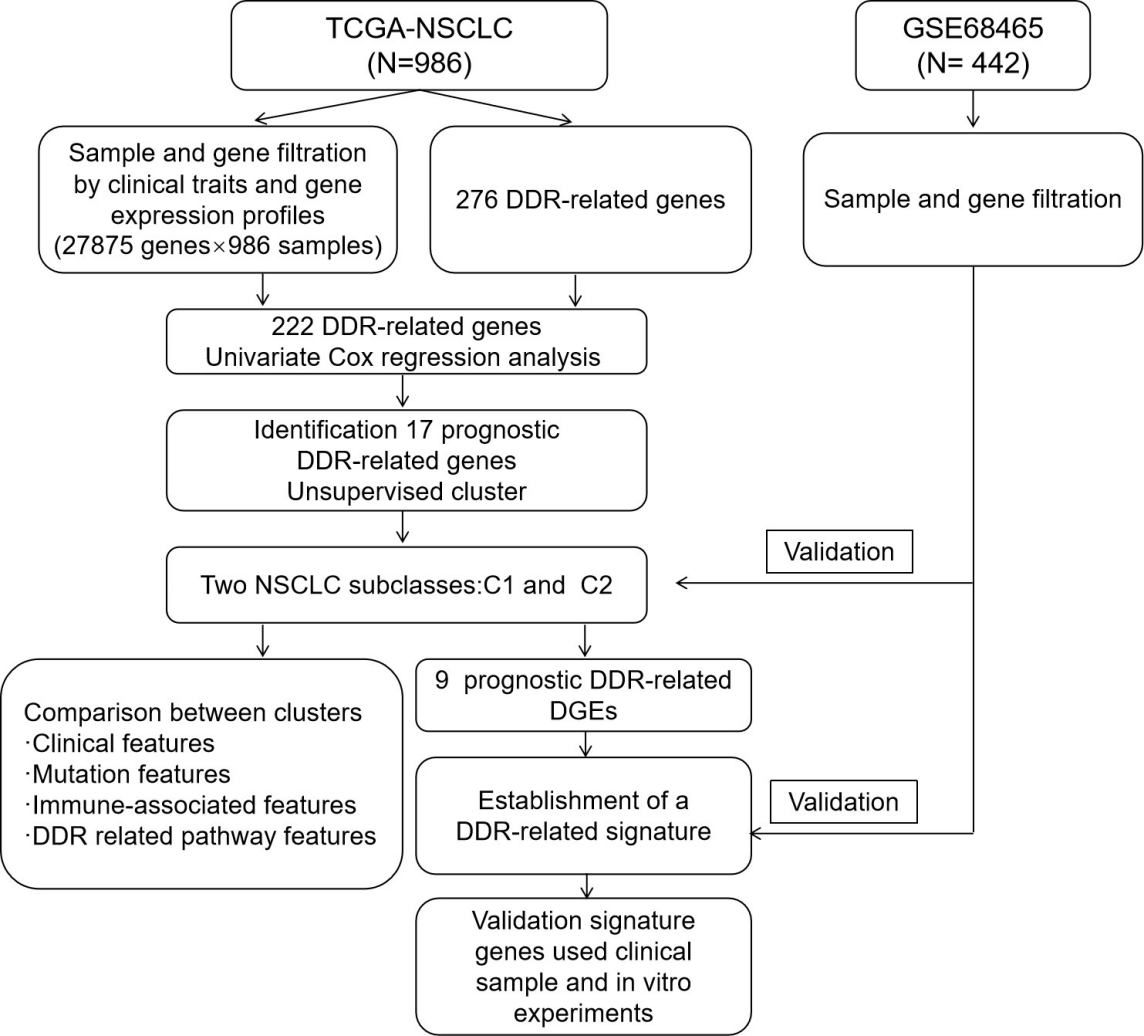
Study flow chart.

The study design is illustrated *via* a flow chart in **Figure 1**, and **Supplementary Table 1** outlines the clinical characteristics of the training and testing sets. In total, 986 NSCLC samples with all clinical characteristics were used as a training set. Two TCGA NSCLC datasets (LUAD and LUSC) gene expression profiles were combined; genes that had undetectable expression or low MAD (NA) in more than 70 percent of the samples were excluded, and the genes were corrected in batches by using the sva package in R. Finally, the expression profiles of 27,875 genes in 986 samples were used for training set. To perform clustering, we acquired matrix of mRNA expression for the 276 initial DDR-relevant genes (**Supplementary Table 3**). Following primary filtering, 222 of the 276 DDR-related genes were expressed in both the training and validation sets. Finally, a total of 222 genes related to DDR were selected for further analysis.

**Figure 2:**
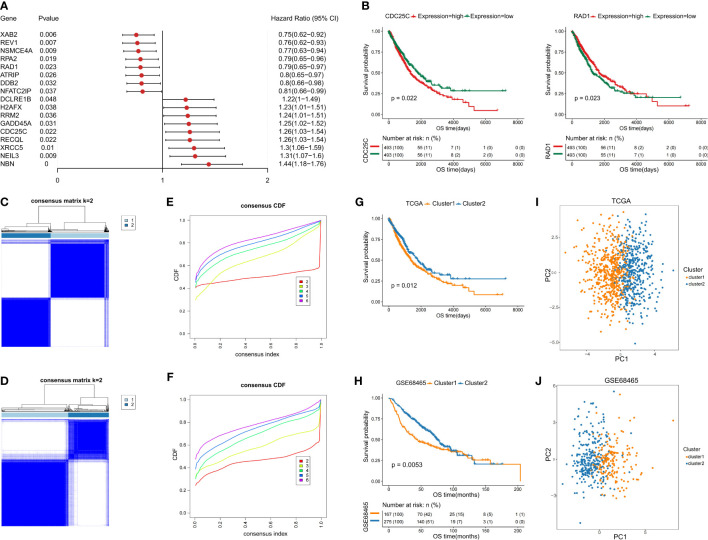
Identification of two NSCLC subclasses based on the prognostic DDR-related genes. **(A)** Forest map of prognostic DDR-related genes. **(B)** Kaplan–Meier (KM) survival curves with transcriptomic profile of prognostic DDR-related gene. **(C, D)** Identification of NSCLC subclasses with PAC clustering using 17 prognostic DDR-related genes in the training and testing sets. **(E, F)** The CDF plot shows a flat middle segment for K = 2 in the training and testing sets. **(G, H)** OS of both subclasses (C1 and C2) in the training and testing sets. **(I, J)** PCA showing the distribution of the two NSCLC subclasses in the training and testing sets.

To identify the prognostic DDR-related genes for classification, the Univariate Cox proportional hazards regression model was conducted, which demonstrated that only 17 of the above DDR genes showed significant effects on survival in the training set (**Figure 2A**). Of the 17 DDR genes found to be related to prognosis of NSCLC patients, high expression of 8 genes (*ATRIP*, *DDB2*, *REV1*, *RPA2*, *NFATC2IP*, *NSMCE4A*, *RAD1*, and *XAB2*) contributed to good overall patient outcome, and high expression of 9 genes (*RECQL*, *RRM2*, *XRCC5*, *CDC25C*, *DCLRE1B*, *GADD45A*, *H2AFX*, *NBN* and *NEIL3*) was indicative of poor prognosis. For better visualization, we generated a forest plot and Kaplan–Meier survival curves using the transcriptomic profiles of the 17 DDR genes to identify the subgroups **(** **Figure 2B** and **Supplementary Figure 1**). To further investigate the potential biological function and mechanism of the 17 prognostic DDR-related genes, we analyzed the 17 potential signature genes by GO and KEGG enrichment analyses. The 17 genes were mostly enriched in nucleotide excision repair, homologous recombination, cell cycle checkpoint process, DNA damage checkpoint, DNA integrity checkpoint, *TP53* signaling biological, non-small cell lung cancer, and cellular response to radiation process or pathways (**Supplementary Figures 2A–D**).

Based on the 17 prognostic DDR-related genes, cluster analysis was performed on all NSCLC samples, which indicated two unsupervised horizontal clusters. The proportion of ambiguous clustering (PAC) method was performed to identify the optimal cluster number (K) of unsupervised clustering (16). Based on the consensus score of the CDF curve, k=2 was identified as the optimal number of clusters (**Figures 2C, E** **)**. Finally, in the training set, 986 NSCLC patients were divided into two clusters, namely, cluster 1 (C1) and cluster 2 (C2), which contained 534 and 452 samples, respectively.

Furthermore, we performed PCA to identify the difference between the two clusters and validate the subclass assignments. It was found that the two clusters were positioned at different corners of the two-dimensional coordinate systems (**Figures 2I, J** **)**. Moreover, we extracted the expression data for the above 17 DDR-related genes and obtained 442 eligible samples in the testing dataset for further analysis. Similarly, k=2 was the optimal number of clusters, NSCLC patients in the testing set were classified into two distinct subclasses, which shared the same distribution as the training set according to PCA and the Z-score method (**Figures 2D, F, J** **)**.

To investigate the differences between the two subclasses, survival analyses were conducted, which demonstrated that C2 had a longer median survival time than C1. The comparison of the training and testing sets for both subclasses indicated that the OS probability was significantly different (p=0.012 and p=0.0053) (**Figures 2G, H** **)**. In addition, the heatmaps indicated that the expression levels of the above selected 17 genes involved in the NSCLC subclass signature showed large differences between the two groups (**Supplementary Figure 2E, F** **)**. Thus, these findings clearly demonstrated that the two subclasses have different molecular and prognostic characteristics.

### Prognostic DDR gene-based NSCLC subtypes show distinct clinical and molecular characteristics

**Figure 3:**
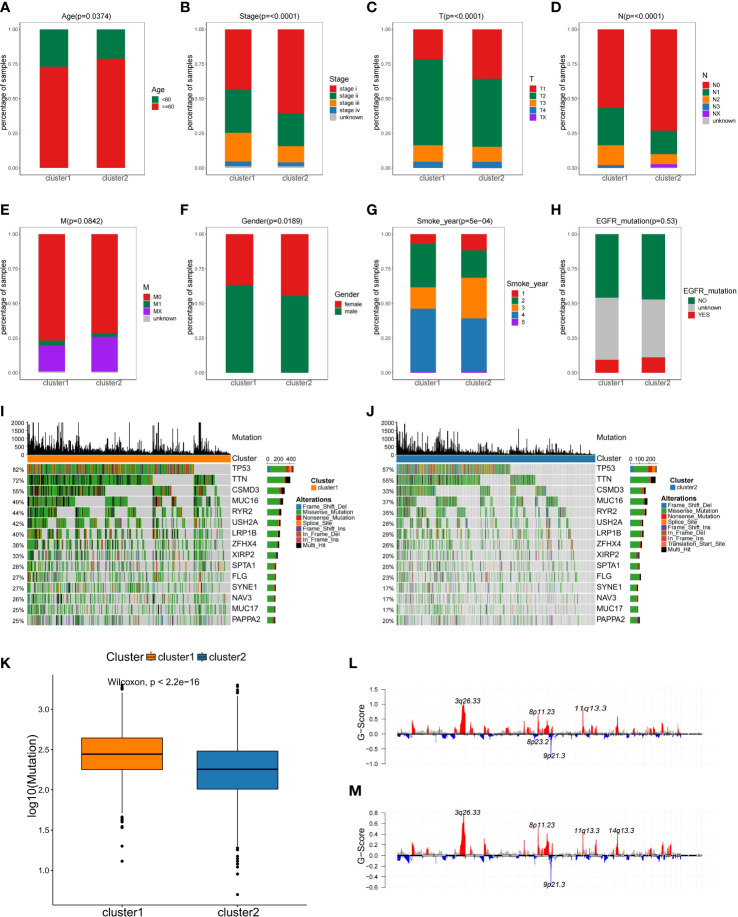
The comparisons of the clinical features between two NSCLC subtypes. **(A–H)** Analysis of clinical features between NSCLC subclasses by Fisher’s exact test. **(I, J)**Comparison of mutation alterations among subtypes in the training set. OncoPrint of mutation status of 15 shared genes among the top 20 in C1 **(I)** and C2 **(J)**. **(K)** Comparison of the absolute mutated number between NSCLC subclasses. Distribution of somatic copy number alteration in C1 **(L)** and C2 **(M)**.

For better discrimination between the two NSCLC subclasses, Fisher’s exact test was used to evaluate the relationship between the clinical features of the two subtypes. Among two subtypes, there were significant differences in age, smoking, gender, T staging, and N staging, but not in M staging and *EGFR* mutation (**Figures 3A–H**).

Gene mutation status is also of vital importance for tumorigenesis (24), disease-free survival (25), drug efficacy (26, 27), and immunotherapy response (28). As part of the analysis of genomic alterations, we also evaluated gene mutation differences. The mutated genes in each subgroup were ranked by mutation frequency. Altogether, 15 overlapping genes were identified between these two subclasses in the top 20 mutation frequency genes, and the median mutation frequency over all patients was plotted for each percentile in C1 and C2, and the proportion of mutations was found to differ in two subtypes (**Figures 3I, J** **)**. For example, the TP53 mutation frequency in C1 subtype was 82%, while in C2 subtype was only 57% (**Figures 3I, J** **)**. Additionally, the C1 subtype exhibited more mutations than C2 subtype (**Figure 3K**). However, there was no difference in copy number variation regions between the two subtypes (**Figures 3L, M** **)**. This observation implied that individual tumor mutation status appeared to be important indexes for personalized therapy for NSCLC patients.

**Figure 4:**
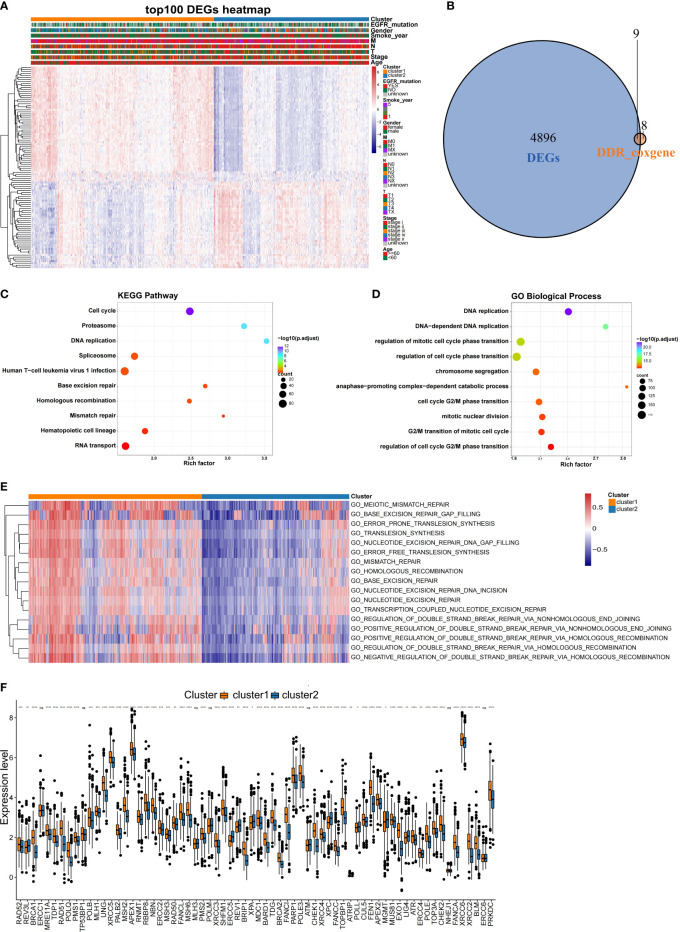
Comparison of the molecular characteristics between the NSCLC subtypes. **(A)** Heatmap of top 100 DEGs expressed in NSCLC subtypes as annotated by clinical features. **(B)** Venn diagram showing the intersection between the prognosis-related DDR genes and DEGs in the training set. **(C)** KEGG and **(D)** GO analysis results of DEGs between the NSCLC subclasses in the training set. **(E)** Comparison of the DDR pathway and key DDR pathway genes between the NSCLC subclasses. Heatmaps were generated to show the biological processes, in which red indicates activation status and blue indicates inhibition status. **(F)** Comparison of expression differences of key DDR genes in the two subclasses. *,P < 0.05; **, P < 0.01; ***, P < 0.001; ****, P < 0.0001; ns, no significance.

Since this was a classification based on DDR related genes, we further explored whether different subtypes have specific biological processes and DDR molecular characteristics. Using the training dataset, the DEGs were identified, and GO analyses were performed. In total, 4905 DEGs (**Supplementary Table 2**) were identified for the two subclasses with a threshold of adjusted P value< 0.05 and |log2FC| > 0.2, and 9 of these DEGs overlapped with the 17 prognostic DDR-related genes (**Figure 4B**). A heatmap was generated to show the top 100 DEGs in each subclass (**Figure 4A**). Genes with a significant difference in expression between the two classes of NSCLC were regarded as subclass-specific genes. GO analysis further revealed that these subclass-specific genes were largely enriched in the cell cycle, DNA replication, base excision repair, homologous recombination, mismatch repair pathways, and biological process, indicating that different activated status among two NSCLC subtypes (**Figures 4C, D** **)**. Then, we further explore whether the molecular characteristics varied between subtypes by GSEA analysis. With a threshold of |log2FC| > 0.3 and adjusted P value< 0.05, a total of 526 significantly differential biological processes were screened for the two subclasses, and 17 of these belonged to DDR pathways (**Figure 4E**). We also performed the Wilcoxon rank-sum test and the 80 key DDR pathway genes have significant differences between the NSCLC subtypes, such as *XRCC5* and *SHFM1* were significantly different between the subtypes (**Figure 4F**). These data demonstrated that the DDR gene-based NSCLC subtypes were characterized by unique clinical and molecular characteristics.

### Prognostic DDR gene-based NSCLC subtypes characterize different immune infiltration

**Figure 5:**
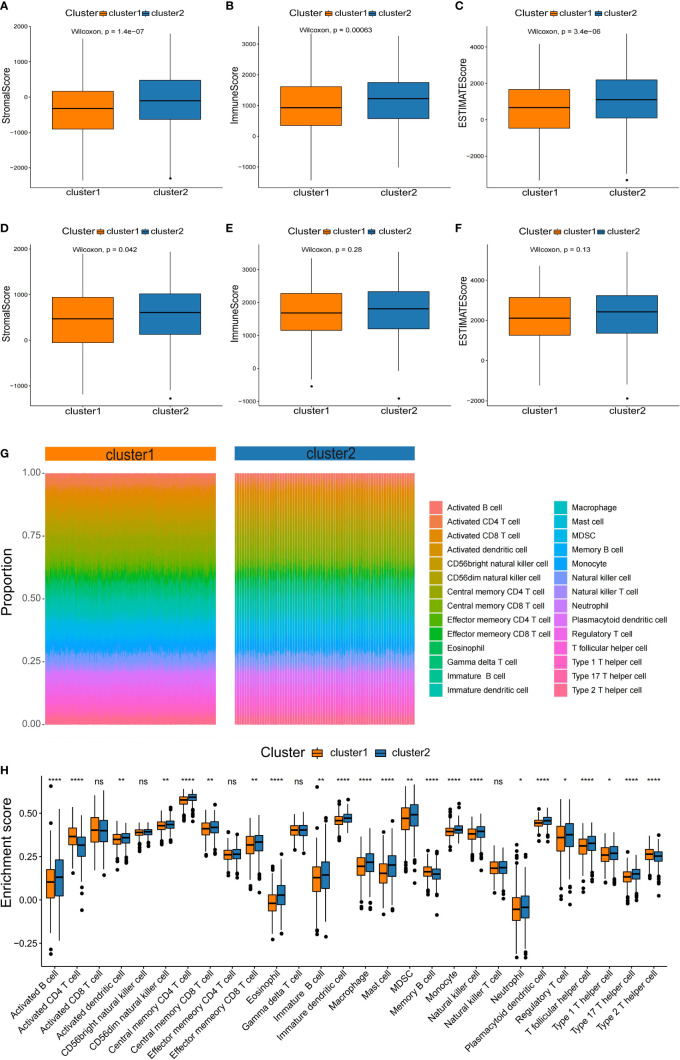
Characterization of the immune infiltration landscape of the two NSCLC subtypes. **(A–F)** Box plots of immune score, stromal score, and total score for NSCLC subclasses derived from ESTIMATE. The lines within boxes on box plots represent median values. The bottom and top lines represent the minimum and maximum values, respectively. **(G)** Heatmap showing the proportion of 28 types of infiltrating immune cells in C1 and C2 by ssGSEA algorithms. **(H)** Comparison of the proportion of 28 types of infiltrating immune cells between the subtypes by the rank sum test. NS denotes P>0.05, * indicates P ≤ 0.05, ** represents P ≤ 0.01, *** indicates P≤ 0.001, and **** indicates P≤0.0001.

It is well known that tumorigenesis and development depend not only on gene mutations, which is also tightly connected with tumor microenvironment (TME). Thus, we compared the differences in immune infiltration between the two NSCLC subtypes. To assess the tumor heterogeneity between the two NSCLC subtypes, ESTIMATE algorithms were used to calculate the stromal score, immune score, and total score (**Figure 5**). It was found that both subtypes had significantly different stromal scores, immune scores, and total scores. The C2 subtype had higher stromal, immune, and total scores than C1 (**Figures 5A** **–C**). According to the results of the testing set, there were significant differences in stromal score between two subtypes (**Figure 5D**), while immune score and total score were not significantly different between subtypes (**Figures 5E, F** **)**. These results were a bit differently in the training set.

Based on the significant differences in immune scores identified between the subclasses, we investigated the immune infiltration landscape of the two NSCLC subclasses. First, the ssGSEA algorithm and rank sum test were conducted to assess the proportion of immune cell infiltration based on the expression levels of 28 types of infiltrating immune cells in the training set (**Figure 5G**). It was found that 23 types of immune cell populations were significantly enriched in both subtypes (**Figure 5H**).

Among the immune cell populations, 20 infiltrating immune cell types, including activated B cells, activated cells, and central memory CD8 T cells, were significantly up-regulated in the C2 subtype compared to the C1 subtype (**Figure 5H**). In contrast, activated CD4 T cells, memory B cells, and type 2 T helper cells were significantly down-regulated in the C2 subtype compared with the C1 subtype (**Figure 5H**).

**Figure 6:**
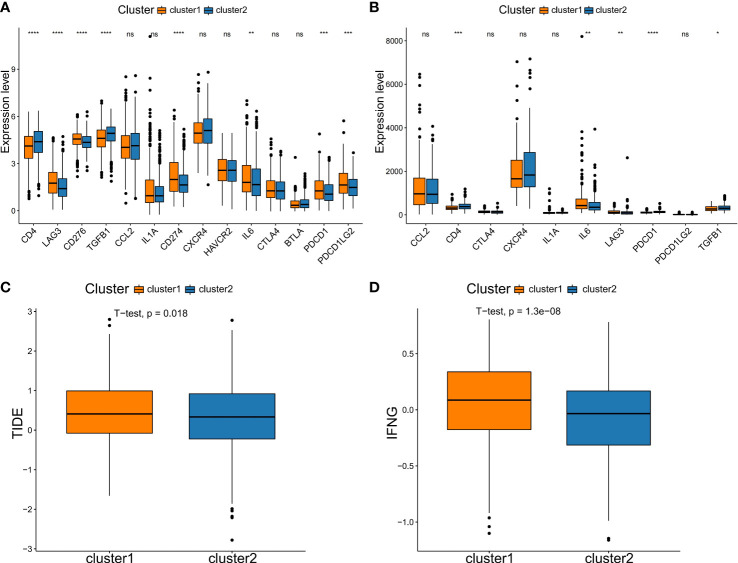
Comparison of immune checkpoint molecules expression and TIDE scores between subtypes. Comparison of the expression of immune checkpoint genes in two subtypes in the training set **(A)** and testing set **(B)**. **(C)** TIDE analysis. **(D)** The expression score of the response prediction biomarker, IFNG, was computed using TIDE analysis. NS denotes P>0.05, * indicates P ≤ 0.05, ** represents P ≤ 0.01, *** indicates P≤ 0.001, and **** indicates P≤0.0001.

In addition, due to checkpoint being molecules are important targets for immune checkpoint inhibitors (ICIs), the immune microenvironment status is a crucial factor in the efficacy and clinical benefit of ICIs in cancers. We next assessed the immunotherapy response in patients in two NSCLC subtypes. Firstly, we evaluated the expression level of several key immune checkpoint molecules between the two subtypes. In the training set, we analyzed the expression of *CD4*, *LAG3*, *CD276*, *TGFB1*, *CCL2*, *IL1A*, *CD274*, *CXCR4*, *HAVCR2*, *IL6*, *CTLA4*, *BTLA*, *PDCD1* and *PDCD1LG2*. Compared to the C2 type, the C1 type had significantly higher levels of immune checkpoint molecules, such as *LAG3*, *CD276*, *CD274*, *IL6*, *PDCD1* and *PDCD1LG2* (**Figure 6A**). The expression of the checkpoint molecules was similar to that in the training set, except for *CCL2*, *CTLA4*, *CXCR4*, *IL1A* and *PDCD1LG2* (**Figure 6B**). Then, the response of ICIs was predicted *via* the TIDE algorithm based on differences in immune infiltration patterns and expression levels of checkpoint molecules. The results showed that C1 had a significantly higher TIDE score compared to C2 (**Figure 6C**). Additionally, we calculated the score of *IFNG*, a CD8 T-cell signature gene, by the TIDE algorithm. Similarly, it was showed that C1 had significantly higher scores compared to C2 (**Figures 6C, D** **)**. Thus, these results suggested that patients in the C2 subtype may responded better to immnotherapy compared to C1 subtype.

### Establishment and validation of a prognostic six DDR-related gene model for NSCLC

**Figure 7:**
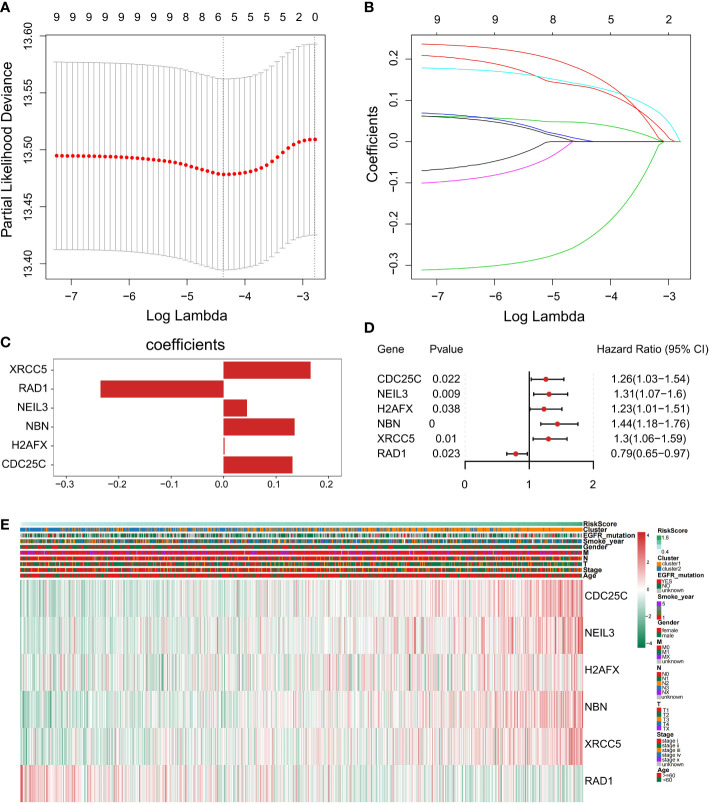
Construction of a prognostic-related signature by the LASSO regression model. **(A)** Selection of the optimal λ-value through a 10-fold cross-validation. **(B)** The fitting processes for LASSO Cox regression models were constructed from the six signature genes. The tuning parameter λ was derived from the partial likelihood deviance with a 10-fold cross-validation, and the coefficient was plotted against Log(λ). The six gene signature was identified based on the best fit profile. **(C)** LASSO coefficient profiles of the six key prognostic DDR-related genes. **(D)** Forest plot of prognostic DDR‐related genes based on univariate Cox regression analysis. **(E)** The heatmap was scaled with the Z-Score using the log2(FPKM+1) expression of signature genes. With the increase in the risk scores, CDC25C, NEIL3, H2AFX, NBN, and XRCC5 expression levels were upregulated and RAD1expression level was decreased.

To construct a genetic signature for clinical application, the most representative genes of each subtype must be identified. Then, the above 9 prognostic DDR-related DEGs (**Figure 4B**) were analyzed by LASSO Cox regression analysis to build a prognostic model. When log(λ)=−4.4, the model exhibited the optimal performance and the fewest number of independent variables (**Figures 7A, B** **)**. This illustrated that the model had optimal performance when it included six prognostic factors. Consequently, the independent prognostic genes *CDC25C*, *NEIL3*, *H2AFX*, *NBN*, *XRCC5* and *RAD1* were chosen to establish a risk score model by LASSO regression analysis. The risk score of each NSCLC patient was calculated as follows: Risk score = (0.131288663692349) × *CDC25C* expression + (0.044719985259839) × *NEIL3* expression + (0.0018724086315065) × *H2AFX* expression + (0.1354895390277) × *NBN* expression + (0.166065772748599) × *XRCC5* expression + (-0.234655329481 268) × *RAD1* expression (**Figure 7C**). Following univariate Cox regression analysis, *CDC25C* [hazard ratio (HR): 1.26, 95% CI: 1.03-1.54, p = 0.022], *NEIL3* (HR: 1.31, 95% CI: 1.07-1.6, p=0.009), *H2AFX* (HR: 1.23, 95% CI: 1.01–1.51, p=0.038), *NBN* (HR: 1.44, 95% CI:1.18-1.76, p=0.00), and *XRCC5* (HR: 1.30, 95% CI: 1.06-1.59, p=0.01) were risk factors for NSCLC prognosis (**Figure 7D**). Moreover, *RAD1* (HR: 0.79, 95% CI: 0.65-0.97, p=0.023) was a protective factor for NSCLC prognosis (**Figure 7D**). Using this model, we further calculated the risk score of each sample and generated a heatmap. With an increased risk score, the expression levels of *CDC25C*, *NEIL3*, *H2AFX*, *NBN* and *XRCC5* increased, indicating that they are high-risk genes; the expression of RAD1 decreased as the risk score increased, indicating that it is a low-risk gene (**Figure 7E**).

**Figure 8:**
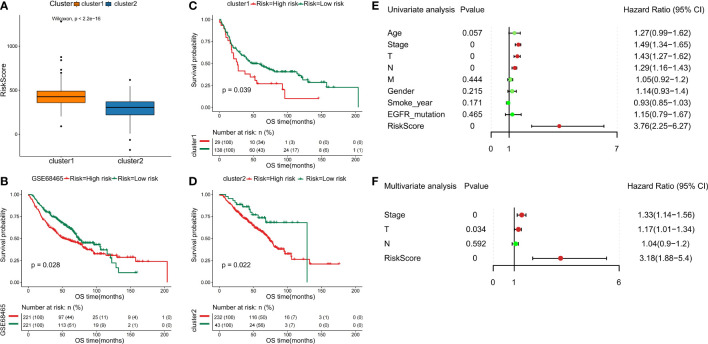
Validation of six DDR-related signatures by the LASSO regression model. **(A)** Distribution of risk scores in NSCLC subclasses. **(B - D)** Kaplan-Meier OS analysis of the prognostic DDR-related signature in NSCLC and NSCLC subtypes. **(E)** Evaluation of the prognostic values for different clinicopathological characteristics (age, smoking_year, gender, stage, T staging, N staging, M staging, and EGFR_mutation) as well as a risk score using univariate Cox regression analysis. **(F)** Multivariate Cox regression analysis was used to test the independence of the risk score and other factors for predicting the prognosis of NSCLC.

Furthermore, we separate the groups as low risk and high risk based on the median-risk score (cutoff = 0.993466). In the training set, the C1 subtype had higher risk scores than the C2 subtype (**Figure 8A**). We observed that patients in the low-risk group had significantly longer OS than those with high-risk scores (**Figures 8B** **–D**). Then, univariate and multivariate Cox analyses were performed to test the performance of the six DDR-related gene model. Firstly, results of univariate Cox analysis showed that the risk score was a risk factor for NSCLC prognosis (HR: 3.76, 95% CI: 2.25-6.27, p=0.000) (**Figure 8E** **)**. Stage (HR: 1.49, 95% CI: 1.34-1.65, p=0.000), T staging (HR: 1.43, 95% CI: 1.27-1.62, p=0.000), and N staging (HR: 1.29, 95% CI: 1.16-1.43, p=0.000) were significantly associated with NSCLC prognosis (**Figure 8E**). Multivariate Cox regression-analysis results showed that the risk score was an independent risk factor for NSCLC (HR: 3.18, 95% CI: 1.88-5.40, p=0.000; **Figure 8F**). Along with the risk score, stage and T staging were also independent prognostic predictors of NSCLC (HR: 1.33, 95% CI: 1.4-1.56, p=0.000; HR: 1.17, 95% CI: 1.01-1.34, p=0.034; **Figure 8F**). Collectively, these data indicated that the six DDR-related signatures could act as an independent prognostic factor for NSCLC, highlighting the importance of the DDR landscape in NSCLC patients.

### Verification of six prognostic DDR-related genes in NSCLC tissues

**Figure 9:**
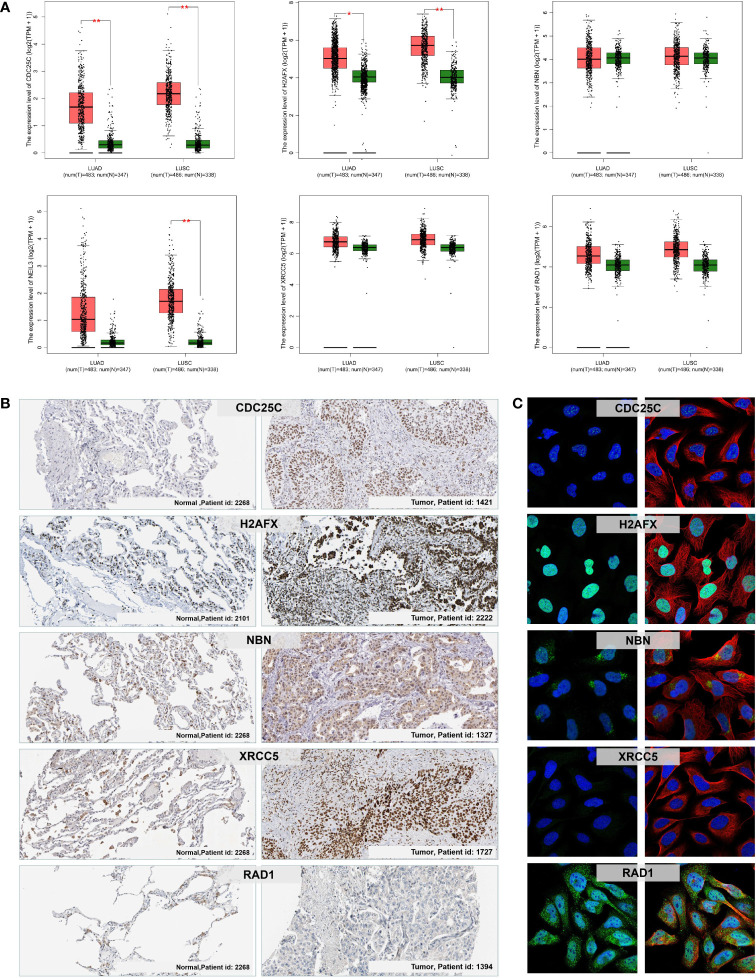
mRNA and protein expression levels of the prognostic DDR-related signature genes in NSCLC. **(A)** The mRNA expression of CDC25C, NEIL3, H2AFX, NBN, XRCC5, and RAD1 in normal lung and NSCLC (LUAD and LUSC) tumor tissues *via* the GEPIA. *p < 0.05; **p < 0.01. **(B)** Immunohistochemistry analysis showing the protein expression of the signature genes in normal lung and NSCLC tumor tissues obtained from the HPA database (data for NEIL3 were not available). **(C)** Immunofluorescence images of CDC25C, H2AFX, NBN, XRCC5, and RAD1 in cells with green, blue, and red indicating target proteins, nuclei, and microtubules, respectively.

We further verified the expression level of the six prognostic DDR-related genes in NSCLC tumor and control normal lung tissues. Firstly, we evaluated the mRNA expression of six DDR-related genes in NSCLC tissues in GEPIA, which demonstrated that *CDC25C*, *NEIL3*, *H2AFX*, *NBN*, *XRCC5* and *RAD1* were all significantly higher expressed in NSCLC *c*ancer tissues compared to with normal lung tissues (**Figure 9A**). Further, we analyzed the protein expression and distribution in clinical tissue specimens from the HPA online databases (www.proteinatlas.org). Immunohistochemistry and immunofluorescence analysis showed that CDC25C, H2AFX, NBN, XRCC5 and RAD1 had positive strong expression in NSCLC tumor tissues and negative weak staining in normal lung tissues (**Figure 9B**) and mainly distributed in nucleus and cytoplasm (**Figure 9C**).

**Figure 10:**
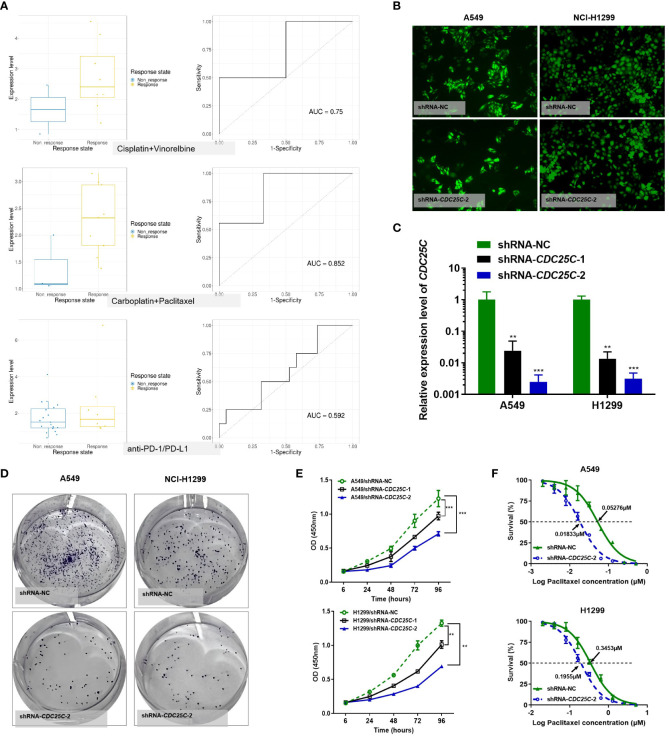
Effects of CDC25C knockdown on proliferation and chemosensitivity in NSCLC cells. **(A)** Drug response analysis by Cancer Treatment Response gene signature DataBase (CTR-DB). **(B)** Immunofluorescence was performed 72 h after transfection. **(C)** Expression of CDC25C mRNA by qRT-PCR in A549 and NCl-H1299 cells transfected with shRNA-NC and shRNA-CDC25C. **(D)** Plate colony formation assay (n=3). **(E)** CCK8 detection of the proliferation of shRNA-CDC25C-transfected A549 and NCl-H1299 cells (n=4). And all data are illustrated as mean ± SD;.NS, no significance; *p<0.05; **p < 0.01; ***p < 0.001; and ****p < 0.0001. **(F)** Drug sensitivity of Paclitaxel was represented by the half-maximal inhibitory concentration (IC50) (n = 4).

Among the above 6 DDR prognostic genes, *CDC25C* was found to have a good predictive effect on the treatment response of lung cancer (**Figure 10A** **)**. As an important cell cycle regulatory protein, *CDC25C* participates in regulating G2/M progression and in mediating DNA damage repair. To better understand the function of six DDR-related gene Model, we selected *CDC25C* to verify its biological function *in vitro*. *CDC25C* expression was significantly reduced by shRNA-*CDC25C* in A549 and NCl-H1299 cells (**Figures 10B, C** **)**. After silencing *CDC25C* expression, cell proliferation ability was significantly inhibited (**Figures 10D, E** **)**. *CDC25C* is a phosphatase family specific cyclin that acts at the G2/M phase of mitosis. We further selected Paclitaxel, a cycle-specific antitumor drug, to analyze the effect of CDC25C expression on chemotherapy reactivity. It showed that decreased *CDC25C* expression resulted in increased Paclitaxel sensitivity of NSCLC cells (**Figure 10F**). These experimental results further demonstrated that CDC25C is a risk gene for NSCLC, it may contribute to the occurrence and development of NSCLC by promoting cell proliferation and affecting drug sensitivity.

## Discussion

Despite progress in the detection and treatments for NSCLC, the prognosis remains poor. Molecular heterogeneity is currently a clinical challenge for NSCLC therapy and prevention. Monitoring and understanding the molecular effects of a defined alteration (e.g., a gene expression alteration, a drug treatment, or a gene mutation) has become key to addressing this challenge. Although some previous studies on molecular typing of lung cancer have provided novel insights into lung cancer precision medicine (29–31), the applicability and accuracy of these indicators for diagnosis, prognosis, and treatment response prediction of lung cancer are not satisfactory. Recently, DDR functional abnormality has been considered a promising anticancer target (32, 33), which prompted us to focus on molecular subtyping in DDR to obtain a better understanding of the molecular changes induced by DDR in the treatment response and prognosis of NSCLC patients.

In the present study, we analyzed the relationship between DDR gene expression and NSCLC prognosis, and we divided NSCLC samples into two subtypes according to prognostic DDR gene expression. Then, constructed a prognosis prediction model of NSCLC, providing a basis for clinical treatment and survival prediction of NSCLC. We first screened 17 genes related to prognosis from 222 DDR-related genes. According to the expression profiles of 17 prognostic DDR-related genes, 986 NSCLC patients in the training set were classified into two distinct DDR-relevant clusters (C1 and C2), and the reproducibility of this subtyping was verified in a testing set. There were significant differences in clinical features, molecular features, immune cell components, gene mutations, and clinical outcomes between the two subtypes. Finally, we constructed a prognostic six DDR-related gene model based on *CDC25C*, *NEIL3*, *H2AFX*, *NBN*, *XRCC5* and *RAD1* expression *via* LASSO Cox regression analysis. In this model, a high risk score was indicative of a poorer prognosis for NSCLC patients. Multivariate Cox regression analysis suggested that the risk score could independently predict survival in NSCLC. Among them, *CDC25C*, *NEIL3*, *H2AFX*, *NBN* and *XRCC5* were found to be risk factors for NSCLC, while *RAD1* was found to be a protective factor for NSCLC.

As is well known, the functional status of DDR have close relationships cancer onset and prognosis and impacts treatment effectiveness. The above 6 DDR prognostic-related genes mainly play roles in DDR by participating in homologous recombination (HR), non-homologous end-joining (NHEJ) and base excision repair (BER) pathways, and these repair pathways are the main types of DNA damage repair. Previous studies have reported that NBN (also called NBS1) is a component of the DNA damage sensing complex MRE11-RAD50-NBS1, playing key roles in HR, NHEJ and BER pathways by binding to ataxia telangiectasia mutated (ATM) kinase (34). Overexpression of NBS1 induces metastasis of cancer cells by activating various pathways (35). Moreover, high levels of NBS1 are associated with poor prognosis and chemotherapy resistance in cancer (36). When ATM DDR pathways is activated, a number of proteins downstream of these kinases are phosphorylated. The signature genes of *CDC25C*, *XRCC5* and *H2AFX* (also called *H2AX*) that we identified are also downstream genes of the DDR pathway. CDC25C is an important cell cycle regulatory protein that participates in the regulation of G2/M progression and the DDR process. Many studies have shown that CDC25C is highly expressed in lung cancer (37, 38). Abnormal expression of *CDC25C* is associated with tumorigenesis, development, metastasis, chemoradiotherapy resistance, and poor prognosis (39–41). XRCC5 (also called Ku80 or Ku86) is an essential component of the NHEJ pathway, and it is highly expressed in lung adenocarcinoma and promotes cisplatin resistance (42, 43). Studies have established that high expression levels of XRCC5 are associated with poor prognosis in LUAD patients (44). However, *XRCC5* knockdown inhibits cell proliferation and increases chemoradiotherapy sensitivity in esophageal squamous cell carcinoma lines (45). As a central component of DDR signaling, ATM phosphorylates histone H2A variant H2AX to generate γ-H2AX, which is considered to be an early indicator for the initiation of DSBs and of DNA damage response. γ-H2AX is used as a biomarker of lung chemoradiotherapy (46–48), and its overexpression is an independent prognostic indicator of poor OS in NSCLC patients (29, 49). Moreover, the γ-H2AX promotes NBS1 expression at damage sites, that it perhaps promotes HR or DNA checkpoint signaling (50). *NEIL3* is acknowledged as a DNA base excision repair enzyme that removes bulky base lesions from DNA, contributes to the choice of the DSB repair pathways by promoting BER (51, 52). It is overexpressed in LUAD, and its overexpression is related to LUAD stage, tumor size, and poor prognosis (53). In LUAD, cell cycle and *TP53* signaling pathways are the two major pathways affected by *NEIL3 (* 53). In addition, several studies have shown that the loss of *NEIL3* reduces cellular proliferation and high expression of NEIL3 can promote chemoradiotherapy resistance (54–57). *RAD1* plays crucial roles in DNA repair and cell cycle checkpoint control; however, the role of *RAD1* in cancer is not completely understood, and there may be differences in its effects in different tumor types. The present study demonstrated that *RAD1* was highly expressed in LUAD, and higher expression of *RAD1* is correlated with poor survival of LUAD patients (58). However, some studies have reported differences regarding the role of *RAD1* in tumors. A previous study has reported that *RAD1* knockdown decreases cell viability and increases cell sensitivity to cisplatin, and they demonstrated that *RAD1* acts as a BRCA-like tumor suppressor in hereditary ovarian cancer (59). Another study has reported that mouse *RAD1* deletion enhances susceptibility for skin tumor development (60). However, the role of these six DDR-related genes linked to NSCLC in tumor development and function remains largely unknown. Then, RT- qPCR and immunohistochemistry analyses demonstrated that these six genes were all activated in NSCLC, which implied that the six signature genes involved in DDR in NSCLC might not benefit prognosis and could even be resistance to the treatment with platinum-containing regimens. Thus, these six genes in the classifier may provide new insights into NSCLC complex etiology, and these findings may also provide important clues in the development of future therapies to treat DDR-based cancers and even a promising therapeutic target in the treatment of malignant tumors.

Like most tumors, lung cancer is a multi-stage development process that involves multiple genes and multiple factors. Furthermore, we found that there were significant differences between the two subtypes in clinical features, molecular features, immune cell components, gene mutations, DDR pathway activation status and clinical outcomes. The high-risk patients (C2 subtype) had more active DDR status, which indicated that activated DDR pathway led to poor prognosis and resistance for chemoradiotherapy. Another hand, a higher proportion of *TP53*, *TTN*, *CSMD3*, *MUC16*, *RYR2*, *USH2A*, *LRP1B*, *ZFHX4*, *XIRP2* and *SPTA1* mutations occur in C1 subtype compared to C2. Like, approximately common 53% of NSCLC patients harbor activating mutations in *TP53* (data from cBioPortal), but the proportion of *TP53* mutations for C1 was up to 82%. Several studies have demonstrated that *TP53* mutations may accelerate the incidences of therapy resistance (61–63). Immune infiltration analysis showed that stromal score, immune score, and total score were significantly higher at high-risk patients. In addition, immune checkpoint genes, including *CD4*, *IL6*, *CD274* (also known as *PD-L1*), *LAG3*, *PDCD1* (also known as *PD1*) and *TGFB1* were higher expressions in the low-risk patients. Further, TIDE analysis demonstrated that the high-risk patients had significantly lower score, implying that high-risk patients may respond better to immunotherapy. These results suggested that the unresponsiveness of the low-risk NSCLC patients to ICB treatment may be caused by PD-1*/*PD-L1 expression or combination with abnormal expression of other genes (64). For example, *TGFβ* attenuates tumor response to *PD-L1* blockade by contributing to exclusion of T cells (65) or other regulatory mechanisms, such as alteration of DDR mutation.

Collectively, we identified six DDR-related prognostic genes, namely, *CDC25C*, *NEIL3*, *H2AFX*, *NBN*, *XRCC5*, and *RAD1*, which may be promising therapeutic targets as well as prognostic markers for NSCLC. According to the prognostic six DDR-related gene model, it suggested that low-risk NSCLC patients choose chemoradiotherapy regimen, while high-risk patients choose immune checkpoint inhibitors for further treatment may have a better prognosis. However, the current study has several limitations. First, the biological functions of *CDC25C*, *NEIL3*, *H2AFX*, *NBN*, *XRCC5* and *RAD1* in NSCLC are not fully understood. Second, the mechanisms of *CDC25C*, *NEIL3*, *H2AFX*, *NBN*, and *XRCC5* serving as risk factors as well as *RAD1* serving as a protective factor for NSCLC prognosis are unclear and will require further study. Third, Prospective clinical studies should be conducted to validate the six DDR-related genes as predictive or prognostic markers in patients with NSCLC.

## Conclusion

In conclusion, we have established a six DDR-related gene signature for prediction of NSCLC prognosis. This signature is independently predictive of NSCLC patient survival. Among these genes, *CDC25C*, *NEIL3*, *H2AFX*, *NBN*, *XRCC5* and *RAD1* were all validated to be upregulated in NSCLC tumor tissues. All signature genes demonstrated a positive association with unfavorable prognosis of NSCLC patients, except for *RAD1*, which was associated with better prognosis. In addition, there were close interactions between the genes, and they regulate both DNA damage response and anticancer efficacy. Therefore, *CDC25C*, *NEIL3*, *H2AFX*, *NBN*, *XRCC5* and *RAD1* may be potential therapeutic markers in NSCLC. According to this risk model, doctors can stratify NSCLC patients by risk score before treatment, thus providing NSCLC patients with individual management and optimal therapeutic strategies.

## Data availability statement

The original contributions presented in the study are included in the article/**Supplementary Material**. Further inquiries can be directed to the corresponding authors.

## Author contributions

LL, F-ZH, and B-JZ conceived and designed the study as well as wrote the manuscript. F-ZH and J-ZZ conducted most of the experiments and data analysis. W-JL, LT, and J-BL provided the scientific research platform and conditions. Z-YS, W-YZ, and S-XL participated in data collection and provided assistance for experiments. All authors reviewed and approved the manuscript.

## Funding

This work was funded by the National Natural Science Foundation of China (No. 81903710 and 81903715), the Science and technology planning project in Zhuhai (No. ZH2202200090HJL), the Hospital Pharmacy Research Foundation of Guangdong Province (No.2022A01), and The Natural Science Foundation of Guangdong Province (No.2022A1515012648).

## Conflict of interest

The authors declare that the research was conducted in the absence of any commercial or financial relationships that could be construed as a potential conflict of interest.

## Publisher’s note

All claims expressed in this article are solely those of the authors and do not necessarily represent those of their affiliated organizations, or those of the publisher, the editors and the reviewers. Any product that may be evaluated in this article, or claim that may be made by its manufacturer, is not guaranteed or endorsed by the publisher.
