# Supplementary figures and images for: A novel DNA damage repair-related signature for predicting prognositc and treatment response in non-small lung cancer
Source: Front Oncol. 2022 Nov 4;12:961274. doi: 10.3389/fonc.2022.961274 (PMC9673481; doi:10.3389/fonc.2022.961274)

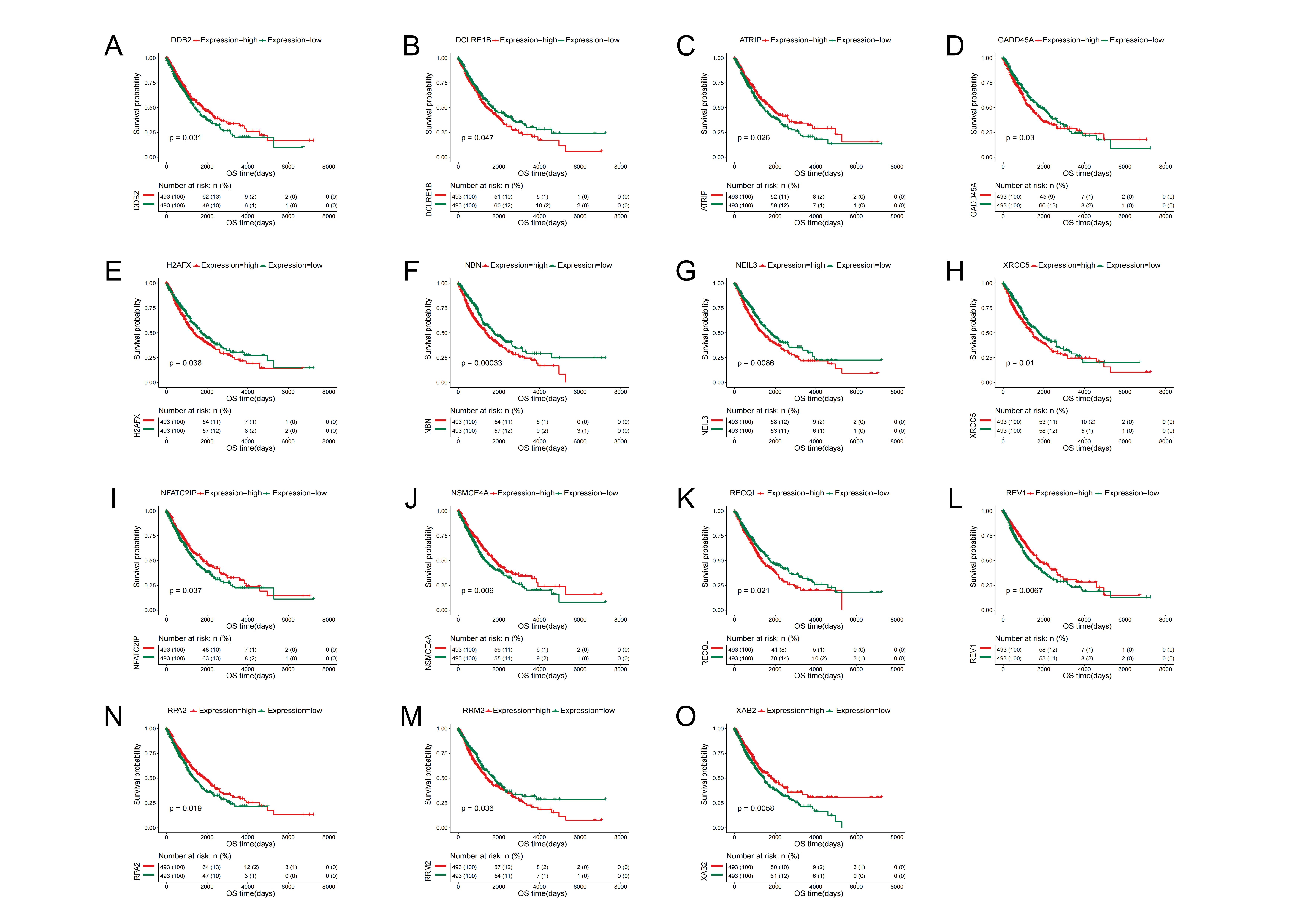

Supplement: Supplementary file 1 [file Image_1.jpeg]

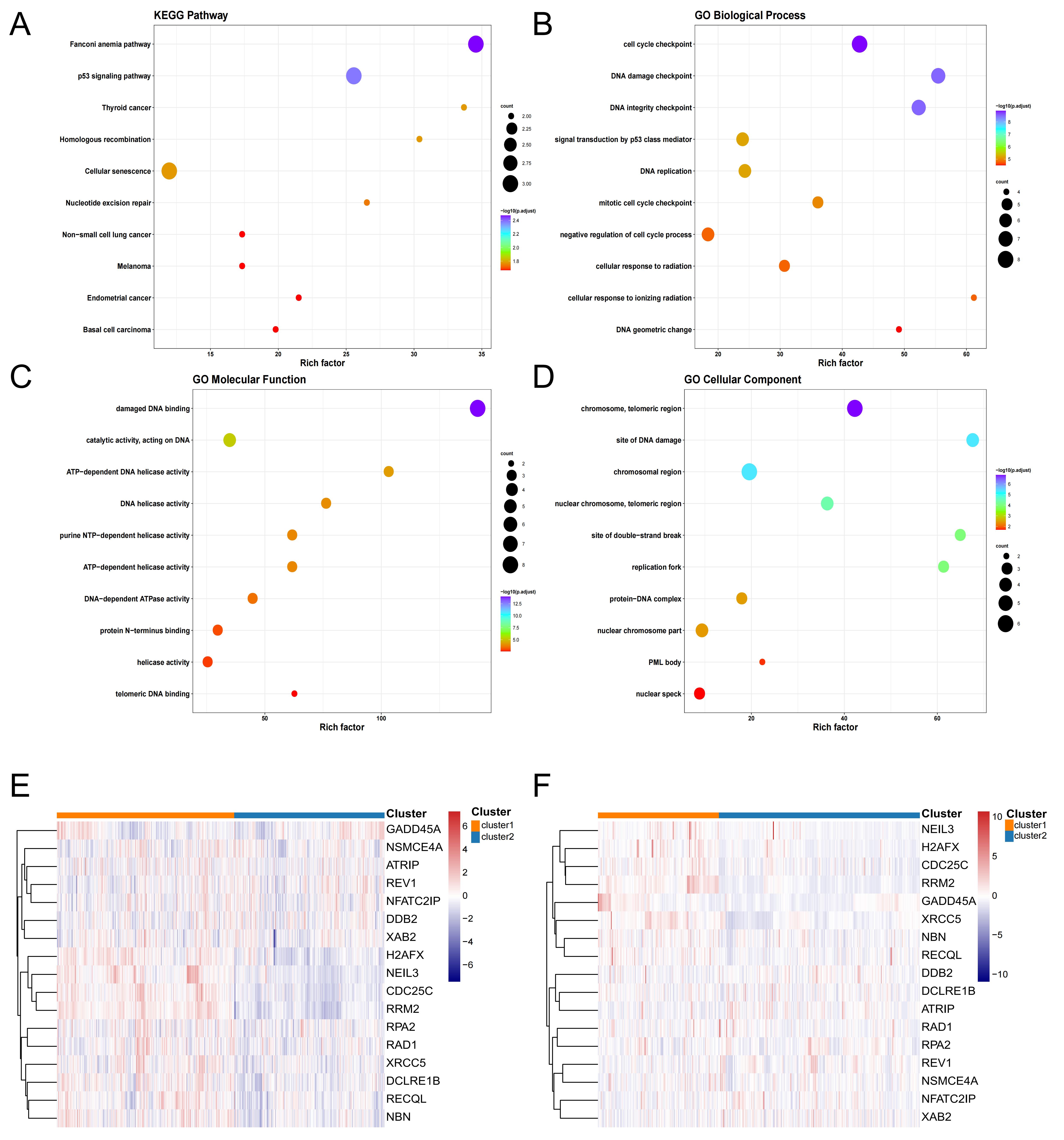

Supplement: Supplementary file 2 [file Image_2.jpeg]
